# Acute epiglottitis caused by COVID-19: A systematic review

**DOI:** 10.17305/bb.2022.8861

**Published:** 2023-08-01

**Authors:** Xiangming Meng, Chengzhou Han, Yangyang Wang

**Affiliations:** 1 Department of Otolaryngology, Wuxi Huishan District People’s Hospital, 2 Zhanqian North Road, Luoshe Town, Huishan District, Wuxi, China

**Keywords:** Coronavirus disease 2019 (COVID-19), severe acute respiratory syndrome coronavirus 2 (SARS-CoV-2), acute epiglottitis (AE), supraglottitis, omicron

## Abstract

The COVID-19 pandemic has caused substantial population infections worldwide. COVID-19 has been reported to cause acute epiglottitis (AE); nonetheless, COVID-19-related AE is poorly understood by healthcare workers because of the disease’s low occurrence. This systematic review aimed to improve knowledge of the clinical characteristics of COVID-19-related AE. We conducted a comprehensive search of the literature databases PubMed, Web of Science, Embase, and Scopus, using various keywords and descriptors, such as “COVID-19,” “SARS-CoV-2,” and “AE” in combination with the AND/OR operator. This review included 11 patients with COVID-19-related AE, all of whom were adults except for one 15-year-old girl. COVID-19-related AE was more prevalent in males, who accounted for 81.8% of patients. Patients with COVID-19-related AE experienced symptoms, such as hoarseness, dysphagia, odynophagia, sore throat, and dyspnea. Hoarseness may be one of the typical symptoms of COVID-19-related AE. Five patients with COVID-19-related AE had coexisting diseases, including hypertension, obesity, diabetes, obstructive sleep apnea, Wolff–Parkinson–White syndrome, and intracranial tumors. Antibiotics and steroids were commonly administered. Five patients with COVID-19-related AE underwent intubation and cricothyroidotomy airway management. Due to the low success rate of intubation, emergency tracheotomy is the recommended option for patients with COVID-19-related AE who present with more severe dyspnea. AE could be an uncommon manifestation of COVID-19, and SARS-CoV-2 infection should be considered as a possible cause of AE. Healthcare workers should be vigilant in recognizing COVID-19-related AE.

## Introduction

Coronavirus disease 2019 (COVID-19) is a newly emerging infectious disease in humans caused by the severe acute respiratory syndrome coronavirus 2 (SARS-CoV-2), a novel RNA beta coronavirus with an enclosed genome [1]. The COVID-19 global pandemic has been ongoing for 3 years, and it has caused a massive number of infections and over 6.8 million deaths, according to the World Health Organization (WHO) [2]. COVID-19 can manifest clinically as fever, cough, dyspnea, fatigue, sore throat, and loss of taste or smell [3, 4]. Furthermore, COVID-19 can cause neurological damage and sudden sensorineural hearing loss [5]. Infection of pregnant women with SARS-CoV-2 can cause hearing impairments in their newborns [6].

Acute epiglottitis (AE), also referred to as supraglottitis, is characterized by inflammation of the epiglottis and associated supraglottic tissues, including the aryepiglottic folds and vallecula [7, 8]. The inflamed epiglottis can swell and cause airway obstruction, leading to severe respiratory distress and even death in severe cases [7, 9]. *Haemophilus influenza* is the most common cause of AE, a hazardous bacterium that causes meningitis, pneumonia, and other potentially fatal diseases [9]. Following the introduction of the *Haemophilus influenza* type b (Hib) vaccine, the incidence of AE in children dramatically reduced [10]. In the vaccine era, streptococci, such as *Streptococcus pneumonia* and Group A *Streptococcus*, are the leading causes of epiglottitis [11]. However, SARS-CoV-2 infection can also cause AE, and thus AE may also be a symptom of COVID-19 [12].

Internationally, Fondaw et al. described the first case of COVID-19 presenting with AE [13]. This patient developed respiratory failure and underwent an emergency cricothyroidotomy and tracheostomy before undergoing successful treatment. Although cases of COVID-19-related AE have been documented worldwide since then, the total number of cases reported thus far is only a dozen. Furthermore, there is a high degree of overlap between the numerous comorbidities of AE and COVID-19 [13]. Given the small number of occurrences of COVID-19-related AE, the knowledge of medical staff about the disease is limited.

The purpose of this systematic review was to improve the understanding of the clinical characteristics of COVID-19-related AE.

## Materials and methods

We conducted a systematic review following the Preferred Reporting Items for Systematic reviews and Meta-Analyses (PRISMA) guidelines [14]. This literature review did not require ethics committee approval.

### Search strategy

We performed a systematic search of the literature databases PubMed, Web of Science, Embase, and Scopus, using a variety of descriptors for “COVID-19,” “SARS-CoV-2,” and “AE” with the AND/OR operator. The studies were not restricted by publication status, language, or publication date. The retrieval strategy relied primarily on a combination of medical subject headings and keywords. The final search was conducted on 14 January 2023. Two reviewers (MX and HC) independently determined the eligibility of the studies and extracted the data.

### Inclusion/exclusion criteria

Original studies or case reports published in the English language on patients with AE who were identified as having COVID-19 by polymerase chain reaction (PCR) or by antibody testing as recommended by WHO were included. Irrelevant articles, non-English language articles, editorials, letters without data, and conference abstracts were excluded.

### Data extraction

Two reviewers (MX and HC) used an Excel (Microsoft Inc., USA) spreadsheet to extract data. The following information was included: author, publication year, country of origin of the patient, time of COVID-19 confirmation, setting, coexisting diseases, symptoms, presence of pneumonia, antibiotics, steroids, and airway management.

## Results

### Search outcomes

**Figure 1.**
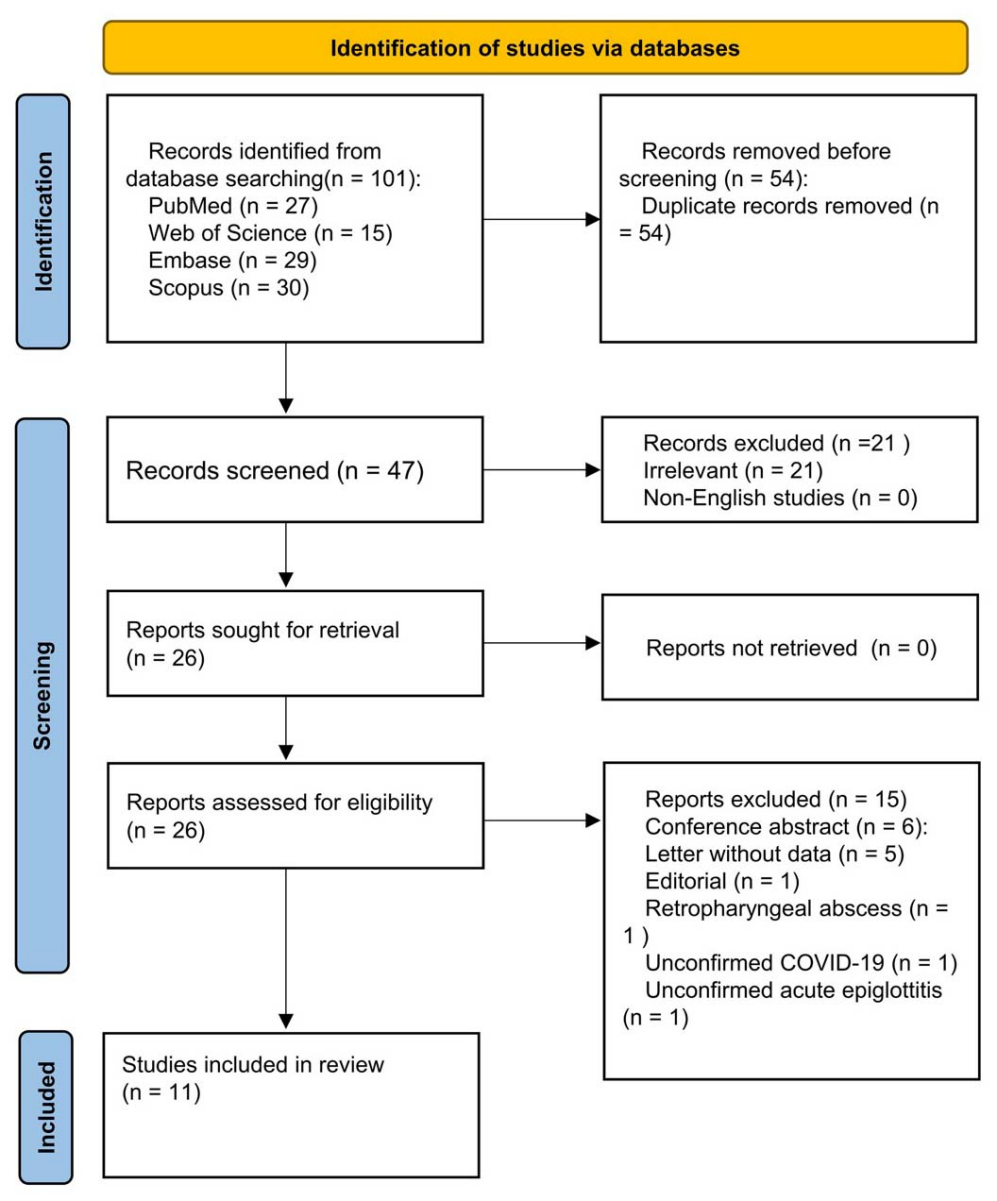
Flowchart of the literature screening process.

We retrieved 101 publications from 4 electronic databases, with 47 remaining after removing duplicates. The literature was then further screened by title and abstract, and 21 irrelevant studies and zero non-English studies were eliminated. By identifying the full-text literature, six conference abstracts without full text, five letters without data, one editorial, one unconfirmed study of AE, one report with unconfirmed COVID-19, and one retropharyngeal abscess study were also excluded. Ultimately, 11 publications met the inclusion criteria and were included in this systematic review. Interestingly, each of the 11 publications had a single patient case report. The characteristics of patients with COVID-19-related AE are summarized in Table 1. A flowchart of the literature search is depicted in Figure 1.

**Table 1:** A summary of studies on COVID-19-related acute epiglottitis

**Authors, year**	**Age, sex**	**COVID-19 confirmation**	**Variants**	**Vaccines**	**Comorbidities**	**Symptoms**	**Pneumonia**	**Antibiotics**	**Steroids**	**Airway management**	**Outcome**
Fondaw et al. [13] 2020	60, M	1 day after AE	Unknown	Unknown	Obesity, hypertension	Dysphagia, hoarseness, shortness of breath, stridor	Yes	Yes	None	Cricothyroidotomy, tracheostomy	Cured
Emberey et al. [15] 2021	53, M	Admission	Unknown	Unknown	None	Odynophagia, neck swelling, shortness of breath	None	Yes	Yes	Tracheostomy	Cured
Nadiger et al. [16] 2021	15, F	Admission	Unknown	Unknown	None	Cephalalgia, hoarseness, dysphagia, globus pharyngeus	Yes	Yes	Yes	None	Cured
Renner et al. [17] 2021	29, M	3 weeks before AE	Unknown	Unknown	None	Respiratory distress, hoarseness	Unknown	Yes	None	Tracheostomy	Cured
Smith et al. [18] 2021	43, M	4 days before AE	Unknown	Unknown	Hypertension	Dysphagia, hoarseness	Unknown	Yes	Yes	Intubation	Cured
Chang et al. [19] 2022	50, M	Admission	Unknown	COVID-19 mRNA	Hypertension, diabetes, obesity, OSA	Fever, sore throat, odynophagia	None	Yes	Yes	None	Cured
Cordia et al. [20] 2022	49, M	Admission	Unknown	Hib	W-P-W syndrome, hypertension	Sore throat, dysphagia	Yes	Yes	Yes	Tracheostomy	Cured
Gezer et al. [12] 2022	58, F	During hospitalization	Unknown	Unknown	Cranial tumors	Shortness of breath, stridor	None	Yes	Yes	None	Cured
Iwamoto et al. [21] 2022	44, M	Admission	N501Y type	Unknown	None	Sore throat, odynophagia	Yes	Yes	Yes	None	Cured
Mitchell et al. [22] 2022	32, M	Admission	Unknown	Hib	None	Sore throat, hoarseness, odynophagia	Unknown	Yes	Yes	None	Cured
Yamada et al. [23] 2022	22, M	1 day before AE	Unknown	Unknown	None	Odynophagia, hoarseness, dysphagia	None	Yes	Yes	None	Cured

M: Male; F: Female; AE: Acute epiglottitis; Hib: *Haemophilus influenza* type b; W-P-W syndrome: Wolff–Parkinson–White syndrome; OSA: Obstructive sleep apnea.

### Countries of patient origin

Patients with COVID-19-related AE originated from five countries. The United States contributed the most patients to this systematic review, with six patients accounting for more than half of the total number of patients included in this analysis [13, 16, 18–20, 22]. In second place was Japan, with two patients [21, 23]. Additionally, there was one patient each from the United Kingdom [15], Finland [17], and Turkey [12].

### Age and sex

All patients were adults except for one 15-year-old child [16]. Adult patients ranged in age between 22 and 60 years. There was a preponderance of male patients, as 9 of the 11 patients were male, accounting for 81.8% of the total number of patients.

### Coexisting diseases

Five adult patients with COVID-19-related AE had coexisting diseases, accounting for half of the adult patients. A patient can simultaneously have multiple coexisting diseases. Four patients had hypertension, which was the most common concomitant condition [13, 18–20]. Two patients exhibited obesity [13, 19]. Diabetes [19], obstructive sleep apnea [19], Wolff–Parkinson–White syndrome [20], and intracranial tumors [20] were also reported.

### Symptoms

**Figure 2.**
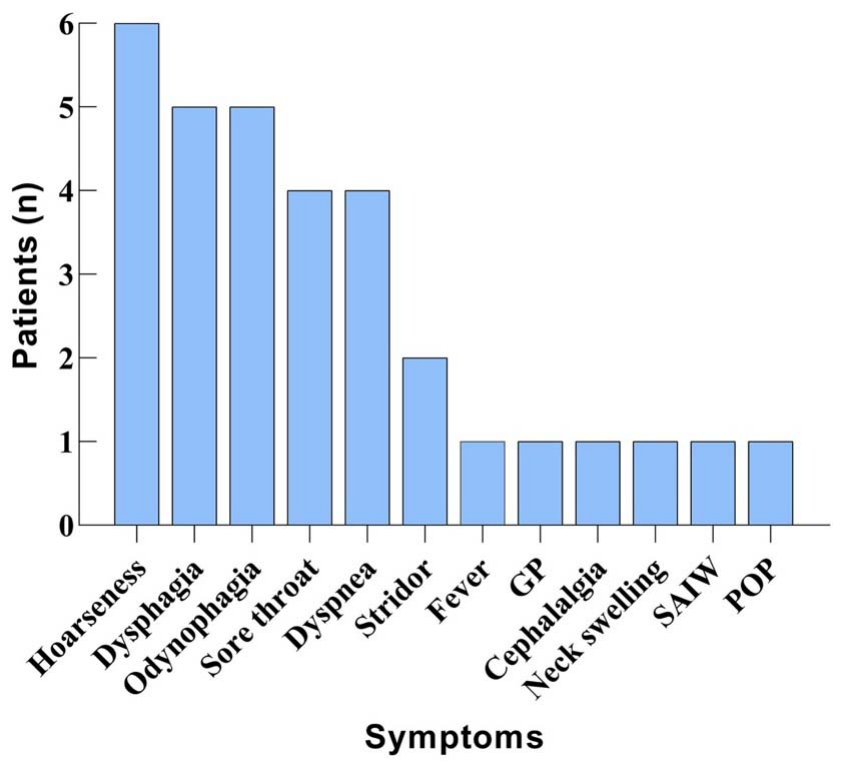
**Distribution characteristics of concomitant symptoms in 11 patients with COVID-19-related acute epiglottitis.** GP: Globus pharyngeus; SAIW: Supraclavicular and intercostal withdrawal; POP: Posterior oropharyngeal pain.

Patients had a variety of multiple simultaneous symptoms, including hoarseness (*n* ═ 6, 54.5%), dysphagia (*n* ═ 5, 45.4%), odynophagia (*n* ═ 5, 45.4%), sore throat (*n* ═ 4, 36.3%), dyspnea (*n* ═ 4, 36.3%), stridor (*n* ═ 2, 18.1%), fever (*n* ═ 1, 9.0%), globus pharyngeus (*n* ═ 1, 9.0%), cephalalgia (*n* ═ 1, 9.0%), neck swelling (*n* ═ 1, 9.0%), supraclavicular and intercostal withdrawal (*n* ═ 1, 9.0%), and posterior oropharyngeal pain (*n* ═ 1, 9.0%). The distribution of characteristic symptoms in patients with COVID-19-related AE is presented in Figure 2. As the figure shows, hoarseness was the most prevalent symptom of COVID-19-related AE.

### Antibiotics

Every patient received antibiotic treatment. Antibiotics administered to these patients included ceftriaxone [15, 16, 18, 22], ampicillin–sulbactam [13, 19, 21], clindamycin [16, 20], azithromycin [13, 16], benzylpenicillin [15], cefuroxime [17], metronidazole [17], moxifloxacin [12], and amoxicillin [22]. Additionally, the type of antibiotic administered in one case was not detailed in the literature [23].

### Steroids

Steroids were administered to 9 of the 11 patients. Dexamethasone [15, 16, 18, 19, 21, 22], hydrocortisone [15, 23], methylprednisolone [12], and prednisone [22] were administered to these patients.

### Airway management

Five patients underwent airway management, representing 45.4% of the total number of patients. Four patients required a tracheotomy to maintain a normal airway [13, 15, 17, 20], one of whom had previously undergone a cricothyroidotomy [13]. Four patients underwent emergency tracheal intubation due to increased respiratory distress [13, 15, 18, 20]; however, only one patient was successfully intubated owing to excessive epiglottis edema and airway narrowing, for a success rate of 25.0% [18].

## Discussion

Based on the existing literature, this systematic review studied the clinical characteristics of COVID-19-related AE, including diagnosis, treatment, and airway management. Despite the enormous number of individuals globally impacted by COVID-19, COVID-19-related AE is uncommon among patients. For this reason, only 11 patients with complete records were included in this systematic review. All 11 patients received antibiotics, 9 patients received glucocorticoids, and 5 patients underwent airway management. Fortunately, all patients were cured.

The onset of COVID-19-related AE may be associated with high expression of angiotensin-converting enzyme 2 (ACE2) and transmembrane protease serine 2 (TMPRSS2) receptors in the epiglottis and a cytokine storm caused by SARS-CoV-2 infection. SARS-CoV-2 is known to infect human cells through ACE2, which stimulates TMPRSS2, thus promoting viral uptake [6]. In one study, ACE2 staining was mild and sparse throughout the laryngeal epithelium, from the epiglottis to the subglottis, whereas TMPRSS2 expression was evident [24]. Nevertheless, interindividual variation in the density of ACE2 receptors exists [25]. Consequently, patients with COVID-19-related AE are likely to have abnormally high levels of ACE2 expressed in the epithelium of the epiglottis, which enhances the invasion of SARS-CoV-2 into the epithelium via the TMPRSS2 receptor to cause illness [18]. Moreover, SARS-CoV-2 infection can contribute to a cytokine storm via repeated epithelial and endothelial damage, resulting in an exaggerated increase in the host’s inflammatory response [15].

COVID-19-related AE in predominantly adult patients may be associated with two factors. First, the introduction of the Hib vaccine has provided some protection against AE, which is commonly caused by *Haemophilus influenza* [10, 26]. Second, most children with COVID-19 were asymptomatic or had mild or moderate symptoms, and the incidence of severe disease in children was 7%, significantly lower than that among adult patients (25.6%) [27]. These observations might be related to the fact that children are less likely to have underlying conditions, such as diabetes, hypertension, or cardiovascular disease [27], resulting in a less destructive situation that does not attack and cause inflammation of the epiglottis.

Acute epiglottitis and COVID-19 share symptoms, such as sore throat and fever, making disease identification and differential diagnosis challenging. Piersiala et al. [28] reported that in the Swedish Omicron wave, some patients with COVID-19 presented to the otolaryngology emergency department with AE-like symptoms, including acute sore throat, severe sore throat, and fever, but laryngoscopy revealed only redness of the hypopharynx and larynx and no epiglottitis edema. Subsequently, Iijima et al. [29] documented an Omicron variant infection in which the patient presented with AE symptoms, including acute sore throat, hoarseness, and a high fever, but with a normal epiglottis on a neck X-ray. Thus, it is necessary for patients with COVID-19 who present with these symptoms of AE to be seen by an otolaryngologist for a definitive diagnosis. Patients may undergo fiberoptic or electronic laryngoscopy, lateral cervical X-rays, and cervical computed tomography. Of these examinations, laryngoscopy is the most straightforward and convenient. For COVID-19 patients admitted to the intensive care unit or isolation ward, bedside video laryngoscopy is an alternative to consider if fiberoptic or electronic laryngoscopy is impractical [30, 31].

Moreover, the 2022 monkeypox outbreak is spreading globally and affecting many people [32, 33]. However, in contrast to earlier monkeypox outbreaks in Africa, the initial symptoms in monkeypox patients in the 2022 outbreak may be pharyngeal, including AE, pharyngitis, and sore throat [33]. Therefore, in the differential diagnosis of this disease, the possibility of human monkeypox should also be considered.

Sore throat and dysphagia are the most typical symptoms of AE [34]. However, hoarseness was the symptom most frequently reported by patients with COVID-19-related AE included in this systematic study. Hoarseness may be one of the characteristic symptoms of patients with COVID-19-related AE, possibly related to the fact that SARS-CoV-2 infection itself can cause mucosal edema in the larynx.

Although most patients with COVID-19-related AE developed the condition after a positive SARS-CoV-2 PCR test, there were some patients with a confirmed diagnosis of COVID-19 after the development of AE. Therefore, in the era of the COVID-19 pandemic, all patients with AE should undergo SARS-CoV-2 PCR testing to exclude COVID-19 [12].

The primary pharmacological treatments for AE involve antibiotics and glucocorticoids. All patients received antibiotic treatment, with ceftriaxone and ampicillin–sulbactam being the most frequently administered antibiotics. Timely and adequate use of antibiotics can effectively control the inflammation of the epiglottis. Since the causative pathogens of these patients cannot be identified, broad-spectrum antibiotics were a suitable choice. Except for two cases, all patients were given glucocorticoid therapy. Glucocorticoids can reduce edema of the epiglottis and minimize the requirement for airway intervention.

Airway management is an essential consideration when treating patients with COVID-19-related AE. Given that SARS-CoV-2 can cause mild to severe airway and laryngeal inflammation, managing the airways of COVID-19 patients is intrinsically difficult [16]. Depending on the severity of the patient’s dyspnea, airway management techniques, such as tracheal intubation, cricothyrotomy, and tracheotomy, may be used to maintain a normal airway. The Katori, Tanaka, and Ovnat-Tamir classifications can all be used to determine if airway interventions are necessary for patients with AE [35]. According to a study from Spain, airway intervention was required for 17% of patients with AE, and epiglottic abscesses, hypersalivation, and smoking may be risk factors [36]. Male sex, dyspnea, stridor, epiglottic edema, aryepiglottic folds edema, elevated C-reactive protein, hyperglycemia, and a history of repeated episodes were identified as features of individuals with high-risk AE in a study conducted in the USA [34].

Adult AE may be affected by underlying systemic comorbidities [37]. Hypertension and diabetes mellitus are the most common systemic diseases, with 34.8% of AE patients suffering from both [37]. Four of the 11 patients with COVID-19-related AE included in this systematic study had hypertension [13, 18–20]. Three of these four patients with COVID-19-related AE who had hypertension underwent airway intervention [13, 18, 20].

Tracheal intubation provides a rapid solution to patients’ ventilation issues. However, only 25% of patients with COVID-19-related AE were successfully intubated. Thus, emergency tracheotomy is the preferred option for patients with COVID-19-related AE who are experiencing more severe respiratory distress. Tracheotomy is an aerosol-generating intervention that raises the risk of SARS-CoV-2 transmission to medical workers [38]. Therefore, when performing tracheotomies, medical workers should follow standard personal protective measures [17].

Interestingly, the four recently reported cases of COVID-19-related AE were treated conservatively with medications without airway intervention [12, 21–23]. This treatment success might be due to the prevalence of Omicron variant, which is significantly less virulent than the original strains and pre-Omicron variants [39].

The limitations of the original studies are also reflected in the limitations of the present systematic review. Because of the low incidence of patients with COVID-19-related AE, each included study was a case report of a single patient. As a result, only 11 patients were included in this systematic review. The different SARS-CoV-2 variants may cause different symptoms of AE. However, only one study recorded the SARS-CoV-2 variant of the patient [21]. The vaccination history of these patients, which includes previous Hib vaccination and COVID-19 vaccination during the pandemic, may affect the occurrence and development of COVID-19-related AE. Of 11 patients included, only 3 had a known vaccination status, with 2 having a previous Hib vaccination history and 1 having received the COVID-19 mRNA vaccine [19, 20, 22]. Furthermore, the ethnic background of many patients was not specified. Therefore, based on the available literature, there is room for improvement in our knowledge of the disease.

Globally, the COVID-19 pandemic is still going strong. It has had disastrous effects on health care in many countries [40]. Based on the models, some experts have predicted that the COVID-19 pandemic will cease in November 2023, but it remains uncertain when it will end [41]. Since China terminated adherence to the zero-COVID-19 policy on 7 December 2022, the number of patients infected with SARS-CoV-2 has risen sharply [42]. Thus, medical workers will continue to combat COVID-19. In clinical practice, medical professionals should be vigilant in recognizing COVID-19-related AE.

## Conclusions

Acute epiglottitis may be a rare manifestation of COVID-19, and SARS-CoV-2 infection should be considered as a possible cause. Hoarseness may be one of the typical signs of COVID-19-related AE. In the era of COVID-19, healthcare workers should be on alert to identify COVID-19-related AE.
